# D-Lactate: Implications for Gastrointestinal Diseases

**DOI:** 10.3390/children10060945

**Published:** 2023-05-26

**Authors:** Barblin Remund, Bahtiyar Yilmaz, Christiane Sokollik

**Affiliations:** 1 Division of Paediatric Gastroenterology, Hepatology and Nutrition, Department of Paediatrics, Inselspital, Bern University Hospital, University of Bern, 3010 Bern, Switzerland; barblin.remund@insel.ch; 2 Department of Visceral Surgery and Medicine, Bern University Hospital, University of Bern, 3010 Bern, Switzerland; bahtiyar.yilmaz@unibe.ch; 3 Maurice Müller Laboratories, Department for Biomedical Research, University of Bern, 3008 Bern, Switzerland

**Keywords:** inflammatory bowel disease, short bowel syndrome, biomarker, intestinal permeability, dysbiosis

## Abstract

D-lactate is produced in very low amounts in human tissues. However, certain bacteria in the human intestine produce D-lactate. In some gastrointestinal diseases, increased bacterial D-lactate production and uptake from the gut into the bloodstream take place. In its extreme, excessive accumulation of D-lactate in humans can lead to potentially life-threatening D-lactic acidosis. This metabolic phenomenon is well described in pediatric patients with short bowel syndrome. Less is known about a subclinical rise in D-lactate. We discuss in this review the pathophysiology of D-lactate in the human body. We cover D-lactic acidosis in patients with short bowel syndrome as well as subclinical elevations of D-lactate in other diseases affecting the gastrointestinal tract. Furthermore, we argue for the potential of D-lactate as a marker of intestinal barrier integrity in the context of dysbiosis. Subsequently, we conclude that there is a research need to establish D-lactate as a minimally invasive biomarker in gastrointestinal diseases.

## 1. Introduction

L-lactate is a familiar molecule to the human body and is also produced in large amounts in human tissues, depending on metabolic conditions [1,2]. In contrast, D-lactate is produced only in minute quantities in human tissues, and is therefore not detectable in the bloodstream under normal physiological conditions [3]. Certain bacteria in the human gut produce D-lactate as a byproduct of carbohydrate fermentation. Lactate-producing bacteria (LAB) are an example of intestinal bacteria, which can generate both L- and D-lactate [4]. Under normal conditions, there is an equilibrium of bacteria and their metabolites, but when the composition of the microbiome is disrupted, such as through a reduction in diversity or an overgrowth of certain bacteria, dysbiosis develops. Dysbiosis is a condition that promotes disease [5] and is a characteristic of several diseases, including inflammatory bowel disease (IBD), metabolic disorders, autoimmune conditions, and psychiatric and neurological illnesses [6,7,8,9]. Elevated intestinal production of D-lactate in dysbiosis can lead to its detection in the bloodstream, and excessive accumulation of D-lactate in the blood can cause metabolic acidosis, also called D-lactic acidosis [10,11]. This metabolic phenomenon is well described as a complication primarily found in pediatric patients with short bowel syndrome (SBS) [12]. It can be life threatening, and therefore, it is crucial to understand the underlying pathophysiology of this metabolic disorder.

Because D-lactate production in the gut increases in the presence of dysbiosis, this review will concentrate on illnesses that affect the gastrointestinal tract. The primary aim of this review is to provide an overview of the sources and metabolism of D-lactate, the pathophysiology of D-lactic acidosis in SBS, and the subclinical rise of D-lactate in diseases affecting the gastrointestinal tract. In addition, the review will address the important question of whether D-lactate could function as a biological marker for intestinal permeability in conjunction with the disease activity of gastrointestinal diseases. It is essential to understand the potential use of D-lactate as a biomarker, as this could have a significant impact on the diagnosis and management of gastrointestinal diseases. This is particularly interesting in pediatrics, with its need for minimally invasive diagnostic tools.

## 2. Materials and Methods

For this narrative review, a search in Pubmed and Clinical Trials.gov was conducted in March 2023. The search terms were “d-lact*”,”gastrointestinal disease*”, “short bowel syndrome”, “dysbiosis”, “microbiome”, “intestinal barrier”, and “biomarker”. These terms were combined into the following query: “d-lact*” AND (“gastrointestinal disease*” OR “short bowel syndrome” OR “dysbiosis” OR “microbiome” OR “intestinal barrier” OR “biomarker”). For analysis, only studies in humans were considered. In total, 193 papers were retrieved. We read the abstracts of the publications and excluded studies that were not relevant to the research question (e.g., discussing other body fluids such as synovial or vaginal fluids, analyzing only fecal D-lactate). The references of the selected publications were reviewed to identify additional relevant articles. In total, 56 papers were included in the review.

## 3. Lactic Acid

### 3.1. Biochemistry

In 1780, the Swedish chemist Scheele discovered lactate, also known as 2-hydroxypropanoate, in sour milk. Lactic acid has two stereoisomers, L- and D-lactic acid. They are enantiomers due to the presence of an asymmetric second carbon atom. Despite their distinct chirality, both enantiomers share similar chemical and physical properties. Lactic acid, at a physiological pH, exists in its conjugate base form (L-lactate for L-lactic acid and D-lactate for D-lactic acid), which does not affect the chirality of the base anion [13].

### 3.2. Sources and Metabolism

#### 3.2.1. Lactate Production and Metabolism

Lactic acid is a byproduct of the process known as anaerobic respiration, and during this process, glucose is broken down into pyruvate in the cytoplasm of cells. Pyruvate can then be converted to lactic acid through the action of lactate dehydrogenases [14]. There are two isomer-specific forms of lactate dehydrogenase (LDH), L-LDH and D-LDH, which produce L-lactate and D-lactate, respectively [15,16,17]. The conversion of pyruvate to lactate (specifically L-lactate form) is an essential process for the body to generate energy during times of oxygen deprivation, such as during intense exercise. Lactic acid can be produced in various tissues, including muscle, red blood cells, and the brain. The accumulation of lactic acid in muscles during exercise can lead to fatigue and soreness. On the other side, lactic acid can serve as a fuel source for other tissues, such as the heart and liver. For this, it is converted back to pyruvate and enters the aerobic respiration pathway to produce energy in the presence of oxygen [1,2,18].

#### 3.2.2. Lactate Production and Metabolism in Humans

Mammalian cells lack the enzyme D-LDH, and therefore produce nearly exclusively L-lactate. However, it has been found that limited amounts of D-lactate are produced endogenously via the methylglyoxal pathway [3]. This pathway generates D-lactate from dihydroxyacetone phosphate (DHAP), an intermediate in various catabolic pathways [19]. It is primarily active in tissues with high rates of glucose utilization, such as the brain and the lens of the eye. In these tissues, the production of D-lactate by the methylglyoxal pathway may serve a protective role by scavenging free radicals and protecting against oxidative stress [20]. The toxic methylglyoxal is quickly converted by the enzymes glyoxalase I and II to D-lactate and glutathione [20]. The created D-lactate is then metabolized in the liver and renal cortex to pyruvate with the mitochondrial enzyme D-2-hydroxy acid dehydrogenase (D-2-HDH) [16,17].

#### 3.2.3. Lactate-Producing and -Utilizing Bacteria in the Gastrointestinal Tract

**Figure 1:**
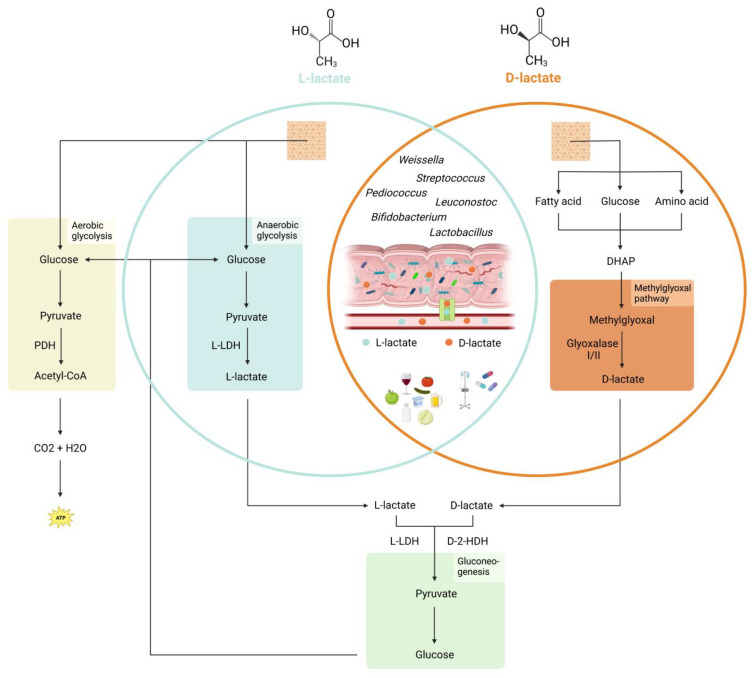
Sources and metabolism of lactate in humans. L-lactate is produced endogenously by anaerobic glycolysis from pyruvate. D-lactate is a product of the methylglyoxal pathway. The precursor methylglyoxal is a byproduct of various catabolic pathways. Further degradation to pyruvate is carried out by the enzymes L-lactate dehydrogenase (L-LDH) for L-lactate and D-2-hydroxy acid dehydrogenase (D-2-HDH) for D-Lactate. Pyruvate can be converted back into glucose by gluconeogenesis. In dysbiosis, lactate-producing bacteria are a significant source of L- and D-lactate. Both can translocate through a disrupted intestinal barrier. L- and D-lactate can also come from external sources, including foods, medications containing propylene glycol, or infusion of Ringer’s lactate solution. PDH = Pyruvate dehydrogenase, L-LDH = L-lactate dehydrogenase, DHAP = Dihydroxyacetone phosphate, D-2-HDH = D-2-hydroxy acid dehydrogenase.

In addition to endogenous production in human tissues, lactate can also be sourced from other places, including gastrointestinal bacteria and exogenous supply (Figure 1). The human gastrointestinal tract is home to millions of bacteria, with various bacterial species capable of synthesizing either L- or D-lactate. The specific type of lactate produced depends on the expression of L-LDH or D-LDH in the bacteria, and some bacteria can also convert one isomer to the other using DL-lactate racemase [4].

Common LAB are *Lactobacillus* (L-lactate, D-lactate, racemic mixture), *Pediococcus* (L-lactate, racemic mixture), *Leuconostoc* (D-lactate), *Weissella* (D-lactate or racemic mixture), *Streptococcus* (L-lactate), and *Bifidobacterium* (L-lactate) [21]. LAB are typically gram-positive, aerobic to facultatively anaerobic, and asporogenous rods and cocci. They are also oxidase-, catalase-, and benzidine-negative, and are unable to utilize lactate. The presence of LAB in the GI tract is beneficial for humans as they help to maintain a healthy gut microbiota, improve nutrient absorption, and stimulate the immune system. Additionally, LAB are often used as probiotics to improve gut health and prevent or treat various gastrointestinal disorders [22]. Pediatric guidelines may recommend probiotics in specific settings, such as acute gastroenteritis and prevention of necrotizing enterocolitis [23].

In addition to LAB, the intestine also contains lactate-utilizing bacteria (LUB), which use lactate as a source of energy. Most LUB belong to the Firmicutes and Actinobacteria phylum [24] and include *Lactobacillus*, *Streptococcus*, and *Bifidobacterium* [25]. *Lactobacillus* and *Bifidobacterium* use the phosphoketolase pathway to convert lactate into short-chain fatty acids (SCFAs) [26], whereas *Streptococcus* use the Embden–Meyerhof–Parnas (EMP) glycolytic pathway [27]. Maintaining a neutral pH in the gut is crucial for these LUB, as they are sensitive to acidic pH levels. The generated SCFAs include butyrate, acetate, and propionate [24]. They are used by the epithelial cells of the colonic mucosa as their main energy source and can provide energy to the body by absorption [28]. Lactate, which is not metabolized to SCFAs, is absorbed by the epithelial cells via the proton-dependent monocarboxylate transporter 1 (MCT-1) [29] or excreted with the stool. By efficiently utilizing lactate, LUB help to maintain the balance of lactate in the intestine, help to reduce inflammation and promote the growth of other beneficial gut bacteria such as Bacteroides and Faecalibacterium, which in turn contribute to overall gut health [30].

#### 3.2.4. Additional Sources

Some fermented foods and beverages such as yogurt, sauerkraut, pickles, sour milk, tomatoes, apples, beer, and wine also contain L- and D-lactic acid [24]. Another source for L- and D-lactate are medications such as ringer lactate solution, sodium lactate, propylene glycol, and some peritoneal dialysate solutions [31].

## 4. D-Lactate in Health and Disease

In healthy individuals, D-lactate is generally considered safe and its amount is negligible. The metabolism of D-lactate is tightly regulated by enzymes and any excess D-lactate is rapidly cleared from the body by the kidneys. The level of D-lactate is typically maintained in balance with the L-lactate level in the body and any changes in the level of D-lactate can indicate an underlying disease condition [32]. Interestingly, when administering D-lactate via oral or IV means to healthy subjects, they do not develop metabolic acidosis or neurological symptoms [12].

### 4.1. D-Lactic Acidosis in Short Bowel Syndrome

D-lactic acidosis (or D-lactate encephalopathy) was first described in 1979 in an adult patient with SBS [33]. D-lactic acidosis is characterized by severe neurological symptoms and metabolic acidosis with a blood D-lactate level of more than 3 mmol/L [34]. Symptoms of D-lactic acidosis include confusion, disorientation, difficulty speaking, and ataxia. In severe cases, D-lactic acidosis can lead to coma or even death. The exact mechanism of D-lactic acidosis in SBS is not fully understood, but it is thought to be related to the increased production of D-lactate by bacteria in the remaining intestine. In individuals with SBS, the remaining intestine may have a reduced capacity to metabolize D-lactate, leading to an accumulation of D-lactate in the blood.

#### 4.1.1. Pathophysiology

SBS is characterized by malabsorption and malnutrition as a result of congenital or secondary loss of a large portion of the small intestine [35]. Because of the resulting altered anatomy, a higher load of undigested or partially digested carbohydrates reach the colon. Fermentation of these carbohydrates to SCFAs by the colonic microbiota leads to a progressive reduction of the intraluminal pH [36]. This pH change supports an overgrowth of acid-resistant, lactic-producing bacteria. In a vicious cycle, these bacteria force a further reduction of pH and thus favor the growth of their own kind [36]. Typically, bacteria of the genus Lactobacillus (L.), such as *L. acidophilus*, *L. fermentum*, *L. buchneri*, *L. plantarum,* or *L. salivarius*, which are all D-lactate producing bacteria, are found in increased concentrations in cases of SBS [10,11]. In summary, in SBS, the increase in undigested carbohydrates in the colon leads to increased SCFA production, which in turn leads to the overgrowth of acid-resistant D-lactate-producing lactobacilli, resulting in an increased accumulation of D-lactate.

A low intestinal pH is a common characteristic in patients with SBS. This leads to a high pH gradient across the epithelial membrane. As D-lactate is co-transported with protons (H^+^) via MCT-1, the uptake of D-lactate from the colon into the blood is enhanced in the setting of SBS [29].

In SBS, the metabolization of D-lactate in the body is impaired due to several mechanisms. First, the low pH in the blood, due to the increased uptake of D-lactate and protons from the colon, inhibits the enzyme D-2-HDH. This inhibition limits the metabolization of D-lactate to pyruvate and enhances the accumulation of D-lactate in the blood [12]. Second, the activity of D-2-HDH appears to be saturable, which at high D-lactate levels results in a build-up of D-lactate [37]. Third, oxalate, a potent inhibitor of the enzyme D-2-HDH, is excessively absorbed in SBS, further impairing the metabolization of D-lactate [38,39]. Last, patients with SBS tend to have higher pyruvate levels, as D-lactate is metabolized to pyruvate via the L-LDH pathway. High pyruvate levels also inhibit the activity of D-2-HDH via a negative feedback loop [40].

D-lactate is partially excreted in the urine. In the renal tubular system, there is a carrier-mediated system (sodium-lactate cotransporter) that can reabsorb D-lactate, so that at low blood levels the urinary excretion of D-lactate is negligible [41]. However, at high blood levels, the kidney is unable to increase the excretion to sufficient amounts for lowering the D-lactate level, even with decreasing reabsorption [41].

#### 4.1.2. Diagnosis

It is important to note that the diagnosis of D-lactic acidosis can be challenging, as it requires measurement of blood D-lactate levels. Unfortunately, in clinical routine, validated D-lactate assays are not available. Additionally, the symptoms of D-lactic acidosis can be similar to those of other neurological conditions, making it difficult to distinguish D-lactic acidosis from other disorders. Lab work typically shows a non-ketotic and non-lactic metabolic acidosis with an increased anion gap in the blood. The urine anion gap may also be increased. In cases of D-lactic acidosis with hyperchloremic acidosis and an increased anion gap in the urine, a misdiagnosis of renal tubular acidosis (RTA) is possible [42,43]. However, analyzing the urine osmolarity gap to calculate excreted NH4+ can help in these cases, as NH4+ excretion is high in D-lactic acidosis but low in RTA [12].

#### 4.1.3. Treatment

Acute management of D-lactic acidosis requires a correction of the acidemia with IV bicarbonate and fluid hydration. Lactated Ringer’s solution contains L- as well as D-lactate and should be avoided [44]. Because carbohydrates build the substrate for D-lactate production with intestinal bacteria, oral carbohydrate intake should be diminished [45]. With hemodialysis, D-lactate can be rapidly cleared from the blood [46]. Antibiotics suppress colonization with D-lactate-producing bacteria. However, caution is required as antibiotics can cause D-lactic acidosis by promoting, e.g., overgrowth of antibiotic resistant D-lactate producing bacteria [47].

Because of the malabsorption of nutrients in SBS, nutritional deficiency may also play a role in the development of neurologic symptoms. Thiamine deficiency was found in a patient with recurrent D-lactate encephalopathy, and after oral thiamine supplementation D-lactic acidosis no longer occurred [48].

Long-term management focuses on preventing recurrences of D-lactic acidosis by correction of the dysbiosis and reestablishment of a healthy microbiome. Replacement of D-lactate-producing bacteria via supplementation of pure L-lactate-producing bacterial species has been successfully employed in pediatric and adult patients [47,49,50]. Another interesting option to change the composition and metabolism of the intestinal microbiota is fecal microbiota transfer, which resulted in resolution of D-lactic acidosis in a case report with a pediatric patient [51].

Nutritional restrictions are beneficial in the long-term management of D-lactic acidosis. Restriction of simple carbohydrate intake reduces the substrate availability for D-lactate-producing bacteria [4]. Oxalate restriction inhibits the enzyme D-2-HDH, which metabolizes D-lactate by converting it to pyruvate [10]. On the other hand, the supplementation of calcium is beneficial, as it increases the intestinal pH and thereby favors the growth of non-acid bacteria and suppresses colonization with lactic acid bacteria [10].

### 4.2. Subclinical Elevations of D-Lactate in the Blood in Different Diseases

#### 4.2.1. Inflammatory Bowel Disease

IBD is a chronic disease characterized by inflammation of the gut accompanied by a dysbiosis and increased intestinal permeability [52]. Recent studies have found significantly higher levels of D-lactate in the blood of IBD patients compared to healthy controls [53,54,55] (Table 1). One study examined adult patients with ulcerative colitis (UC) who received treatment with mesalazine or mesalazine plus rifaximin. All patients had significantly higher D-lactate levels before therapy than after therapy and the decrease in D-lactate levels correlated with a decrease in clinical activity (Mayo Score) and systemic inflammatory markers (C-reactive protein and erythrocyte sedimentation rate) [53]. Interestingly, in the group with co-treatment, this effect was pronounced [53]. A general effect of treatment on D-lactate levels could be confirmed in another study, which included adult UC and Crohn’s disease (CD) patients [54]. A study in CD patients which compared patients with active disease and patients in remission showed that D-lactate was able to be used to discriminate between both disease status (AUC 0.815, 95%CI 0.692–0.904) [55]. From these studies in IBD, an association of blood D-lactate concentration and intestinal inflammation can be hypothesized. Additionally, D-lactate levels may be a useful biomarker for assessing disease activity as well as the effectiveness of IBD treatment. Further research is needed to better understand the relationship between D-lactate and IBD as well as the potential clinical applications of D-lactate measurement in IBD management. Whether D-lactate even plays an active role in the pathogenesis of IBD needs to be determined.

#### 4.2.2. Acute Appendicitis

Acute appendicitis is thought to result from luminal obstruction leading to mucus retention and bacterial overgrowth. The increase in tension of the appendiceal wall accompanied by decreased blood and lymph flow eventually leads to necrosis and perforation [56]. The diagnosis of acute appendicitis is often still challenging, despite improved diagnostic strategies and broadly available ultrasonography. Therefore, different studies have investigated whether D-lactate could be a diagnostic tool in acute appendicitis [57,58,59,60,61]. In all but one study, D-lactate levels contributed to the diagnosis of acute appendicitis. One study with pediatric patients further postulated that D-lactate levels can be used to differentiate between types of appendicitis (e.g., acute vs. perforated) [57]. However, this was not confirmed in two other studies [58,59]. It could be argued that a very localized inflammatory process is not sufficient to raise the blood D-lactate level, and instead, a more spread-out sequence of events is required. Further studies are needed to provide a more conclusive picture of the role of D-lactate in acute appendicitis.

#### 4.2.3. Intestinal Ischemia

Intestinal ischemia is characterized by insufficient oxygenation of the intestinal mucosa, which leads to epithelial damage with increased risk of bacterial translocation. To test its diagnostic and prognostic value, D-lactate was measured in intestinal ischemia due to embolic events [62], sepsis or septic shock [63,64], and complications of ruptured abdominal aortic surgery [65,66]. In all these scenarios, D-lactate was significantly elevated in comparison to controls. It is noteworthy that patients undergoing surgery for mesenteric ischemia showed significantly higher levels of D-lactate in their blood compared to patients undergoing surgery for acute abdomen without intestinal ischemia [63]. In septic patients, D-lactate levels in the blood positively correlated with splanchnic luminal CO_2_ production [63]. From these findings, it can be postulated that hypoxia seems to be a crucial anchor point in the vicious cycle of D-lactate production and translocation. Hypoxia can disrupt the normal metabolism of carbohydrates and could lead to an increase in the production of D-lactate by bacteria, as is known for the production of L-lactate [67]. Furthermore, hypoxia can impair the intestinal barrier function [62,68], resulting in increased translocation of D-lactate and other bacterial metabolites from the gut into the bloodstream. These events can trigger an inflammatory response and further exacerbate dysbiosis, feeding the vicious cycle.

#### 4.2.4. Liver Disease

A dysfunctional gut–liver axis, understood as a situation of crosstalk between the gut microbiome, its metabolites, the immune system, and the liver, seems to play an important role in the pathogenesis of fatty liver disease, alcoholic liver disease, and liver cirrhosis. Dysbiosis and impaired intestinal barrier function are hallmarks of its dysfunction [69]. Accordingly, in patients with liver diseases, including metabolic fatty liver disease, alcoholic liver disease, and liver cirrhosis, increased D-lactate levels in the blood were found [70,71,72,73]. The severity of cirrhosis was not univocally reflected in the D-lactate levels, which may be due to different definitions of patient groups. For example, in one study, D-lactate levels in alcoholic liver disease patients with Child–Pugh A or B cirrhosis were less raised than with acute hepatitis [72]. In another study, there was no difference in D-lactate levels between stable cirrhosis and acute decompensation [68]. A study in hepatitis B patients showed a positive correlation of D-lactate with increasing cirrhosis categorized by Child–Pugh A, B, and C [71]. Furthermore, in metabolic fatty liver disease, D-lactate levels correlated with fatty infiltrations of the liver in ultrasonography. Thereby, D-lactate could be used to distinguish between mild and moderate/severe steatosis [73]. A high disease burden in liver disease is associated with impaired intestinal barrier function due to altered intestinal blood flow as well as a compromised immune response, which leads to dysbiosis, and could thus explain raised D-lactate levels in the blood.

#### 4.2.5. Cystic Fibrosis

Cystic fibrosis (CF) is a monogenetic disease with mutations in the cystic fibrosis transmembrane conductance regulator (CFTR) gene leading to a paucity or absence of the chloride channel activity in epithelial cells. In patients with CF, intestinal dysbiosis is often present, caused by multiple factors including pancreatic insufficiency, decreased gut motility, altered intestinal mucus layer composition, and recurrent treatments with antibiotics [74,75]. We found one study investigating D-lactate in pediatric patients with CF [76]. D-lactate levels correlated with clinical activity, defined by the Shwachman–Kulczycki score and pancreatic insufficiency. There was no association with diet composition or malnutrition, but there was a trend toward higher D-lactate levels in patients with signs of intestinal inflammation expressed by higher fecal calprotectin levels. Further studies are needed to explore whether D-lactate could be a valuable surrogate marker for intestinal health in CF patients.

#### 4.2.6. Diabetes Mellitus as an Example of a Non-Primary Gastrointestinal Disease

Several studies showed significantly increased D-lactate levels in patients with type 1 and type 2 diabetes mellitus (DM) [3,77,78,79,80,81,82,83]. It is postulated that the increased D-lactate in DM patients may be intrinsically caused by higher endogenous D-lactate production. In favor of this hypothesis is the finding that DM patients show higher methylglyoxal levels compared to controls [79,82,83,84]. Methylglyoxal is the only substrate that is metabolized to D-lactate in human cells [85], and high substrate availability could be one factor to explain high D-lactate levels in DM [3,82,86]. Additionally, one study showed that administration of high doses of metformin significantly decreased methylglyoxal levels in parallel with an increase in D-lactate [79]. The authors argue that metformin activates the methylglyoxal pathway, and thus increases degradation of methylglyoxal to D-lactate [79].

A known complication in patients with DM is ketoacidosis. In some cases, the extent of the metabolic acidosis cannot be explained by the measured concentrations of ketones [80,81]. This distinctive feature of ketoacidosis may actually be explained by high D-lactate levels. Ketone bodies are degraded to methylglyoxal via different pathways, including methylperoxidase [85] and methylglyoxal, to D-lactate. Whether dysbiosis and increased intestinal D-lactate production may coexist as causes for measurable D-lactate was not investigated in these studies. However, patients with DM can show a dysbiosis [87,88] and an impaired intestinal barrier [89]. Therefore, it is conceivable that the altered microbiome and the higher intestinal permeability could also cause elevated D-lactate levels in the blood of patients with DM. Unfortunately; studies investigating this hypothesis are not available yet.

**Table 1:** Blood D-lactate levels in mmol/L in different diseases. D-lactate was measured with different laboratory assays.

Disease	Frist Author	Study Group	Blood D-Lactate Levels [mmol/L]
IBD	Yang [53]	Group A (mesalazine)	
		Before treatment	7.18 ± 0.77
		After treatment	5.48 ± 0.63
		Group B (mesalazine + rifaximin)	
		Before treatment	7.22 ± 0.87
		After treatment	3.22 ± 0.38
	Song [54]	UC	0.07 ± 0.03
		CD	0.07 ± 0.03
		Controls	0.01 ± 0.01
	Cai [55]	Active CD	0.18 ± 0.05
		Remission	0.12 ± 0.04
		Controls	0.11 ± 0.04
Appendicitis	Demircan [57] *	Perforated appendicitis	0.44 ± 0.08
		Non-perforated appendicitis	0.16 ± 0.04
		Controls	0.05 ± 0.02
	Caglayan [58]	Edematous appendicitis	0.72 ± 0.54
		Flegmaneous appendicitis	0.72 ± 0.56
		Perforated appendicitis	0.43 ± 0.14
		Controls	0.01 ± 0.01
	Filiz [59]	Acute appendicitis	0.53 ± 0.02
		Perforated acute appendicitis	0.61 ± 0.02
		Nonspecific abdominal pain	0.20 ± 0.01
		Acute abdomen other than appendicitis	0.19 ± 0.01
		Controls	0.18 ± 0.01
	Duzgun [60]	Edematous appendicitis	1.12 ± 0.5
		Flegmaneous appendicitis	1.77 ± 1.3
		Gangrenous appendicitis	1.65 ± 1.0
		No appendicitis	0.40 ± 0.30
	Kwan [61] *	Definitive appendicitis	0.3 ± 0.4
		No definitive appendicitis	0.3 ± 0.5
Intestinal ischemia	Murray [62]	Mesenteric ischemia	0.36 ± 0.04
		Bowel obstruction	0.12 ± 0.02
		Acute abdomen	0.11 ± 0.04
		Controls	0.05 ± 0.01
	Poeze [63]	Septic survivors	
		Admission	0.09 ± 0.11
		24 h	0.11 ± 0.11
		Septic nonsurvivors	
		Admission	0.11 ± 0.14
		24 h	0.24 ± 0.12
	Jorgensen [64]	Septic survivors, mean [range]	0.3 [0–0.6]
		Septic nonsurvivors, mean [range]	0.4 [0.1–0.7]
		Controls, mean [range]	0.03 [0–0.13]
Liver disease	Ruan [71]	Liver cirrhosis	0.15 ± 0.1
		Controls	0.01 ± 0.01
	Zhang [73]	Without MAFLD, median (IQR)	0.15 (0.09–0.19)
		With MAFLD, median (IQR)	0.26 (0.2–0.39)
Cystic fibrosis	Wiecek [76] *	Cystic fibrosis with pancreatic insufficiency, median (IQR)	12 × 10^−3^ (8–13 × 10^−3^)
		Cystic fibrosis without pancreatic insufficiency, median (IQR)	8 × 10^−3^ (7–10 × 10^−3^)
Diabetes mellitus	Talasniemi [3]	DM	40 × 10^−3^ ± 24 × 10^−3^
	Hasegawa [77]	DM	28 × 10^−3^ ± 4 × 10^−3^
		Controls	13 × 10^−3^ ± 1 × 10^−3^
	McLellan [78]	T1DM and T2DM	20 × 10^−3^ ± 1 × 10^−3^
		Controls	11 × 10^−3^ ± 1 × 10^−3^
	Beisswenger [79]	T2DM without metformin	10 × 10^−3^ ± 4 × 10^−3^
		T2DM with metformin ≤1 g/day	14 × 10^−3^ ± 8 × 10^−3^
		T2DM with metformin >1 g/day	13 × 10^−3^ ± 5 × 10^−3^
		Controls	8 × 10^−3^ ± 3 × 10^−3^
	McLellan [83]	T1DM, median (range)	18 × 10^−3^ (6–57 × 10^−3^)
		T2DM, median (range)	20 × 10^−3^ (3–48 × 10^−3^)
		Controls, median (range)	10 ± 4 × 10^−3^ (2–20 × 10^−3^)
	Forni [80]	Diabetic ketoacidosis	0.16 ± 0.07
		Controls	0.04 ± 0.02
	Lu [81]	Diabetic ketoacidosis	3.82 ± 2.5
		DM	0.47 ± 0.55
		Controls	0.25 ± 0.35

* Study with pediatric patients. Values are mean ± SD unless otherwise indicated. UC = ulcerative colitis, CD = Crohn’s disease, MAFLD = metabolic dysfunction associated fatty liver disease, IQR = interquartile range, T1DM = type 1 diabetes mellitus, T2DM = type 2 diabetes mellitus.

## 5. Emerging Role of D-Lactate as a Biomarker

Intestinal permeability refers to the ability of the intestinal lining to allow or prevent the passage of substances from the lumen of the gut into the bloodstream. Increased intestinal permeability has been associated with various gastrointestinal diseases, including inflammatory bowel disease [52,90], intestinal ischemia [91], advanced liver cirrhosis [92,93], and type 1 [94] and type 2 [89] diabetes mellitus. These diseases share pathophysiological similarities, including dysbiosis, with a possible overgrowth of D-lactate-producing bacteria, which displace the healthy gut microbiota [54,72,74,76,87]. Dysbiosis also contributes to the impairment of the intestinal barrier [95], which then allows translocation of D-lactate into the bloodstream [55,62,71,96]. Overall, it can be postulated that, in addition to increased bacterial D-lactate production, an alteration of the intestinal barrier is required to increase blood D-lactate levels in these diseases. Therefore, D-lactate could serve as a useful biomarker for the integrity of the intestinal barrier in the context of dysbiosis.

In certain diseases that lead to elevated D-lactate in the blood, a correlation with disease activity could be discovered. In patients with IBD, lower values were measurable during therapy than before therapy [54]. Patients with acute decompensated liver cirrhosis showed higher D-lactate levels in the blood than patients with stable cirrhosis [72]. D-lactate levels in blood also correlated positively with disease activity in pediatric patients with cystic fibrosis [76]. Assuming that D-lactate is a marker of intestinal barrier integrity, it is reasonable that in situations in which disease activity, and thus impairment of the intestinal barrier, changes, D-lactate levels reflect these altered statuses. D-lactate could therefore be a marker of disease activity in chronic diseases affecting the gastrointestinal tract.

## 6. Limitation of the Study

The studies reviewed here primarily describe preliminary data on small numbers of patients. In addition, the available studies focus on associations with crude outcomes and have a limited granularity. All studies concerning subclinical D-lactate levels were—with the exception of two in acute appendicitis [57,61] and one in CF [76]—performed on adults. More comprehensive data for the pediatric age group are available in SBS, but mostly from case reports.

Unfortunately, no causal relationships and pathomechanisms are explored, such as the determination of the intestinal microbiome to detect D-lactate-producing bacteria or the correlation between the intestinal microbiome and blood D-lactate levels. From a technical point of view, there are no established cutoffs and normal values. The reasons for this are the unavailability of a standard assay and that the determination of D-lactate is still reserved for research settings.

## 7. Conclusions

The presented studies indicate that D-lactate could be a promising biomarker for assessing gut permeability in the presence of dysbiosis. Dysbiosis often coexists with impaired gut barrier function, leading to increased translocation of microbial products into the bloodstream and causing systemic inflammation. Since D-lactate is primarily produced by intestinal bacteria and can only be detected in the blood when the intestinal barrier function is compromised, measuring D-lactate levels could provide valuable information on the status of the gut barrier.

In addition, D-lactate could also serve as an activity marker in chronic diseases that involve dysbiosis and impaired gut barrier function. For example, in inflammatory bowel disease, the severity of the disease is closely related to the degree of dysbiosis and gut barrier dysfunction. Therefore, monitoring D-lactate levels could potentially provide a useful measure of disease activity.

However, further research with larger patient populations, including pediatric patients, is needed to validate the utility of D-lactate as a gut permeability or disease activity marker in clinical practice. In particular, a reliable and standardized D-lactate assay needs to be developed and validated for routine clinical use. If successful, the use of D-lactate as a minimal invasive biomarker could have important implications for the diagnosis, monitoring, and treatment of pediatric diseases associated with dysbiosis and gut barrier dysfunction.

## Abbreviations

CD	Crohn’s disease
CF	Cystic fibrosis
CFTR	Cystic fibrosis transmembrane conductance regulator
D-2-HDH	D-2-hydroxy acid dehydrogenase
D-LDH	D-lactate dehydrogenase
DHAP	Dihydroxyacetone phosphate
DM	Diabetes mellitus
L.	Lactobacillus
LAB	Lactic acid bacteria
LUB	Lactate utilizing bacteria
L-LDH	L-lactate dehydrogenase
LDH	Lactate dehydrogenase
IBD	Inflammatory bowel disease
IQR	Interquartile range
MAFLD	Metabolic dysfunction associated fatty liver disease
MCT-1	Monocarboxylate transporter 1
PDH	Pyruvate dehydrogenase
RTA	Renal tubular acidosis
SBS	Short bowel syndrome
SCFA	Short chain fatty acid
SD	Standard deviation
T1DM	Type 1 diabetes mellitus
T2DM	Type 2 diabetes mellitus
UC	Ulcerative colitis
